# Disruption of mediator complex subunit 19 (Med19) inhibits cell growth and migration in tongue cancer

**DOI:** 10.1186/1477-7819-11-116

**Published:** 2013-05-27

**Authors:** Li-Jun Zhu, Wang-Xiang Yan, Zhong-Wei Chen, Yu Chen, Dan Chen, Tong-Han Zhang, Gui-Qing Liao

**Affiliations:** 1 Department of Oral and Maxillofacial Surgery, Guanghua College of Stomatology, Sun Yat-Sen University, 56 Lingyuanxi Road, Guangzhou 510055, China; 2 Department of Oral and Maxillofacial Surgery, Guangdong Academy of Medical Sciences/Guangdong General Hospital, 106 Zhongshan 2nd Rd, Guangzhou 510080, China; 3 Department of Oral and Maxillofacial Surgery, First Affiliated Hospital, Sun Yat-Sen University, 58 Zhongshan 2nd Rd, Guangzhou 510055, China

**Keywords:** Med19, Tongue cancer, Tumorigenicity, Proliferation, Migration

## Abstract

**Background:**

Mediator complex subunit 19 (Med19) is a critical subunit of the mediator complex that forms a bridge between the transcription factors and RNA polymerase II. Although it has been reported that Med19 plays an important role in stabilizing the whole mediator complex, its biological importance in tongue cancer cell proliferation and migration has not been addressed.

**Methods:**

By using MTT, BrdU incorporation, colony formation, flow cytometric, tumorigenesis and transwell assays, We tested the Med19 role on tongue cancer cell growth and migration.

**Results:**

We demonstrated that lentivirus-mediated Med19 knockdown could arrest tongue cancer cells at G1 phase, inhibit tongue cancer cell proliferation and migration *in vitro*. The tumorigenicity of Med19 short hairpin RNA (shRNA)-expressing lentivirus infected tongue cancer cells were decreased after inoculating into nude mice.

**Conclusions:**

These results indicate that Med19 plays an important role in tongue cancer proliferation and migration, and suggest possible applications for tongue cancer therapy.

## Background

Oral squamous cell carcinoma represents the sixth most common cancer around the world [1], and tongue carcinoma is one of the most common types of oral cancer [2]. Similar to other malignancies, the formation of tongue carcinoma is a complex process associated with accumulation of genetic and epigenetic changes that occur during the progression of the disease. Understanding the molecular mechanisms underlying tongue carcinoma progression may provide unique strategies for the development of molecular-targeted therapies for prevention and treatment of tongue carcinoma. The Mediator complex is a large multiprotein complex which is vital for transcriptional regulation and controls cell proliferation and differentiation [3]. It is expressed ubiquitously and conserved from yeast to mammals [4]. In yeast, Mediator is composed of 25 subunits, and divided into three discrete domains: the head, middle, and tail modules [5-7]. *Med19 (ROX3)* [Ensembl: ENSG00000156603.10] was first identified during a screen for mutants with increased expression of the heme-regulated *CYC7* gene and proposed to be an transcriptional regulator because of its nuclear localization [4]. The hypothesis of Med19 (ROX3) as a transcriptional regulator was further confirmed in a gene expression microarray study in *Med19* deletion strain because a broad range of genes was found to be up- or downregulated after Med19 functional disruption. Furthermore, Med19 has been demonstrated to be a component of the Mediator complex [8] and is essential for mediator binding and its activation of RNA Pol II [9,10]. Structural analysis showed that Med19 is involved in head-module subunits in mammalian mediator complex and plays an important role in the whole mediator stabilization.

The potent function of Med19 as a transcription coactivator for regulating gene expression pattern suggests its role in the development of malignancies. Recently, Med19 was reported to promote the proliferation of bladder cancer, hepatocellular carcinomas, prostate cancer, gastric cancer and breast cancer cells [11-15]. However, the functional role of Med19 in tongue cancer cell growth and migration has not been reported.

In the present study, we constructed recombinant lentivirus delivering short hairpin RNA (shRNA) against *Med19*, which expresses GFP as a marker. The effect of *Med19* silencing in tongue cancer cell proliferation, tumor formation and cell migration was investigated *in vitro* and *in vivo*. Our investigation may gain more insights into the progression of tongue cancer and provide a new target for gene therapy for this lethal disease.

## Methods

### Cell culture

Tongue cancer Tca8113 cells and HEK293T cells were purchased from ATCC and maintained in DMEM supplemented with 10% FBS, at 37°C, 5% CO_2_ (Gibco, Carlsbad, CA, USA).

### Med*19* siRNA infection

Med19 siRNA (5′-AAGGTGAAGGAGAAGCTAAGT-3′) or negative control siRNA (5′- TTCTCCGAACGTGTCACGT-3′) were inserted into pGCSIL-GFP lentiviral vector, respectively. The siRNA plasmids were transfected together into HEK293T cells with lentiviral helper plasmid*s* to generate the respective lentiviruses using Lipofectamine 2000 (Invitrogen, Grand Island, NY 14072, USA). Viral stocks were made and used to infect tongue cancer cells. Cells were collected for mRNA and protein levels detection after 72 h after infection.

### Reverse transcription polymerase chain reaction

Total mRNA samples of tongue cancer cells were prepared with Trizol reagent (Invitrogen, Grand Island, NY 14072, USA) according to the manufacturer’s instructions. Samples (2.0 μg) were used as templates to perform the RT-PCR assay using M-MLV-RTase (Promega, Madison, WI 53711, USA). The resulting cDNA was amplified by using the SYBR-Green Master PCR Mix (Applied Biosystem, Grand Island, NY 14072, USA) in triplicates. Real-time PCR was performed on the TP800 qPCR System (Takara, SW, Akron, OH 44314, USA). Primers used for real-time PCR were as follows: Actin-forward, 5′-GGCGGCACCACCATGTACCCT-3′, Actin-reverse, 5′-AGGGGCCGGACTCGTCATACT-3′; Med19-forward, 5′-GTAACTTCCTGCCTGACCTG-3′, Med19-reverse, TGTGCTTGTGCTTATTCTTCTTC-3′.

### Western blot analysis

Cells were lysed in 1 × SDS lysis buffer (1 M Tris-HCl pH 6.8, 2% SDS, 20% glycerol, 1 mM aprotinin, 1 mM PMSF and 10 μg/mL leupeptin). The protein samples were separated by electrophoresis in SDS-PAGE and then transferred to a polyvinylidene difluoride (PVDF) membrane. After blocking with Tris buffer saline (TBS) containing 5% nonfat milk and 0.1% Tween 20 overnight, the membrane was subsequently incubated with primary antibodies at room temperature for 2 h or at 4°C overnight and with secondary antibody for another 2 h, respectively. The membrane was then developed using the ECL+plus™ Western blotting system (Amersham).

### Cell proliferation assay

Tca8113 cells were infected with Med19 siRNA lentivirus or control lentivirus for 3 days. About 2,000 cells were seeded into each well in 96-well plates. An MTT cell proliferation assay was performed for 5 consecutive days and a BrdU incorporation assay was performed at 24 h and 48 h. Results were expressed as the absorbance at 570 nm and 490 nm, respectively.

### Colony formation assay

Med19 siRNA lentivirus or mock control infected Tca8113 cells were collected 3 days after lentivirus infection. For the plate clone forming experiment, 500 cells were mixed in culture medium, and seeded in 6-well plates and each with three duplicate wells. Afterward, the cells were incubated at 37°C in air with 5% CO_2_ and the media were renewed every 3 days. Two weeks later, the colonies were stained with Giemsa and the colony number was statistically analyzed.

### Cell cycle analysis

Lentivirus infected tongue cancer cells were fixed with 70% pre-chilled ethanol at 4°C for 1 h after 3 days of lentivirus infection. The fixed cells were washed and stained with propidium iodide (PI) mixture containing 50 μg/mL PI and 100 μg/mL ribonuclease in PBS for 45 min at 37°C. The cells were passed through a 300-mesh nylon net before the DNA content was determined by quantitative flow cytometry with standard optics of FACScan flow cytometer (Becton-Dickinson FACSCalibur, San Jose, California, USA). All the groups were performed in triplet and statistically analyzed.

### Transwell migration assay

To explore the role of Med19 in tongue cancer cells, we performed a transwell assay in a 24-well culture plate using the Cell Invasion Assay Kit. (Invitrogen, Grand Island, NY 14072, USA) Briefly, 1 × 10^4^ cells from different groups were seeded on a fibronectin-coated polycarbonate membrane insert (6.5 mm in diameter with 8.0 μm pores) in a transwell apparatus (Costar, Cambridge, MA, USA) and cultured in DMEM. FBS was added to the lower chamber. After incubation for 12 h at 37°C in a CO_2_ incubator, the cells on the top surface of the insert were removed by wiping with a cotton swab. Cells that migrated to the bottom surface of the insert were fixed with methanol, stained with crystal violet, and scored visually in 5 random fields using a light microscope.

### Xenograft model of tumor growth *in vivo*

The study protocol was approved by the institutional ethics board of the Guanghua College of Stomatology, Sun Yat-Sen University. All efforts were made to minimize mice suffering. BALB/c nude mice (male, 4-6 weeks old, 20 ± 2 g) were purchased from Shanghai SLRC Experimental Animal Center. (Shanghai, P.R. China) Cells were resuspended at 2 × 10^7^ cells/mL and an aliquot of 0.25 mL cell suspension was injected subcutaneously into athymic nude mice. Tumor volumes were determined by external measurements and calculated according to the equation, *V* = (*L* × *W*^2^) × 0.52 (V = volume, L = length and W = width) [16]. Mice were sacrificed after 22 days and tumor weights were measured.

### Statistical analysis

All experiments were performed three times in triplicates. The data were analyzed with one-way ANOVA. Differences were considered statistically significant at *P* value <0.05.

## Results

### Transduction of Med19 shRNA into human tongue cancer cells

**Figure 1:**
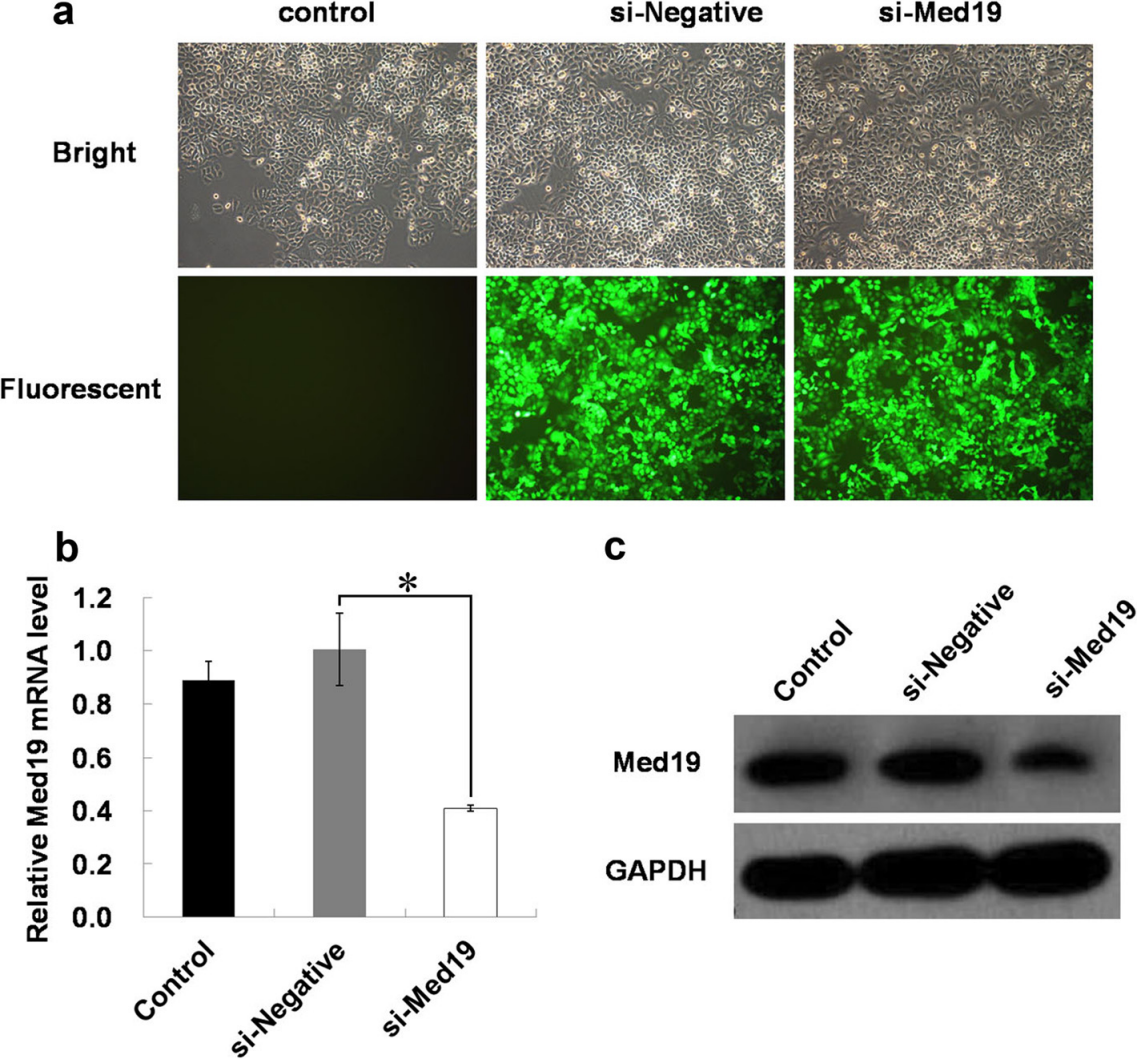
**Med19 shRNA leads to down-regulation of Med19 in tongue cancer cells.** (**a**) Blank Control (Control), nonsense siRNA (si-negtive) and Med19 shRNA (si-Med19) were transduced into tongue cancer cells. (**b**) The mRNA level of *Med19* was downregulated by Med19 shRNA in tongue cancer cells. (**c**) The protein level of Med19 was downregulated by Med19 shRNA in tongue cancer cells.

Recombined lentivirus expressing Med19 shRNA (si-Med19) or negative control shRNA (si-Negative) was transduced into human tongue cancer cells at the multiplicity of infection (MOI) of 100, and more than 90% of infected cells expressed GFP as determined with fluorescence microscopy 3 days post-transduction (Figure 1a). To determine the effect of RNAi on the expression of Med19 in tongue cancer cells, the mRNA and protein levels of Med19 were analyzed after 3 days of lentivirus infection, respectively. The cells transduced with Med19 shRNA showed lower expression of Med19 mRNA and protein than those with negative control shRNA or the blank control cells (Figure 1b, c).

### Proliferation of tongue cancer cells is inhibited by Med19 shRNA

**Figure 2:**
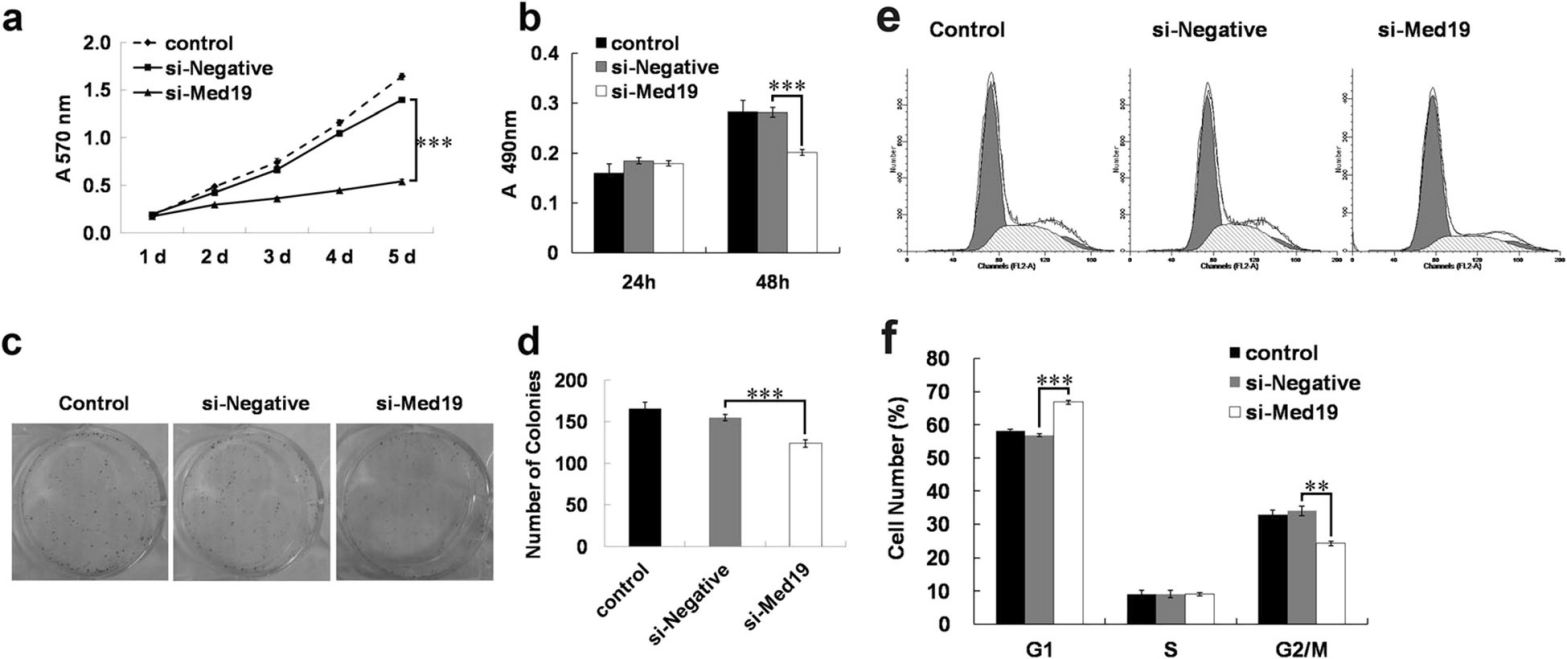
**The proliferation of tongue cancer cells is inhibited upon Med19 shRNA treatment.** (**a**) Growth curve by MTT assay. Cells were treated with negative control shRNA or Med19 shRNA and cultured for one to five days. (**b**) BrdU incorporation assay was performed on cells treated with Med19 shRNA or negative control shRNA. (**c**) Representative colony of tongue cancer cells treated with control shRNA or Med19 shRNA. (**d**) Cells treated with Med19 shRNA made less clone number compared with control shRNA or blank control. (**e**) Fluorescence-activated cell sorting (FACS) analysis of cells phase of tongue cancer cells treated with negative control or Med19 shRNA. (**f**) Cells treated with Med19 shRNA showed higher cell numbers at the G1 phase. Mean values are derived from three independent experiments. Errors indicate standard deviation.

We then detected whether Med19 shRNA affected the proliferation ability of tongue cancer cells by MTT cell proliferation assay and BrdU incorporation assay. As shown in Figure 2a, the growth pattern changed visibly from the second day to the fifth day in tongue cancer cells. Cells treated with Med19 shRNA-expressing lentivirus showed lower growth rate compared with the negative control shRNA or the blank control. To test whether silencing of Med19 could impede cell cycle progression, the incorporation rate of a proliferative marker, BrdU, in scrambled or Med19 siRNA-transduced cells was measured. Consistently, the BrdU assay also showed the growth of cells was inhibited 48 h after the cells were treated with Med19 shRNA-expressing lentivirus compared with negative control shRNA or blank controls (Figure 2b). These results revealed that knockdown of Med19 by shRNA in tongue cancer cells did have an inhibitory effect on cell growth and proliferation.

### The colony formation ability of tongue cancer cells is inhibited by Med19 shRNA

We then further analyzed the proliferation potency of tongue cancer cells treatment with Med19 shRNA by cell colony formation assay. The colony number of Med19 shRNA treated cells decreased from 166.0 ± 7.8 colonies/well in the control group to 124.0 ± 4.4 colonies/well in the Med19 shRNA lentivirus infected group in tongue cancer cells (Figure 2c, d).

### The cell cycle of tongue cancer cells is inhibited by Med19 shRNA

We then performed flow cytometry assay to detect weather Med19 shRNA affected tongue cancer cell cycle by FACScalibur flow cytometer 72 h after transduction. The results showed that the proportion of tongue cancer cells in G1 phase increased significantly with the descrease of cells in the G2/M phase (Figure 2e, f). Our results thus suggested that Med19 shRNA inhibited tongue cancer cells proliferation through modulating cell cycle progression.

### The migration of tongue cancer cells is inhibited by Med19 shRNA

**Figure 3:**
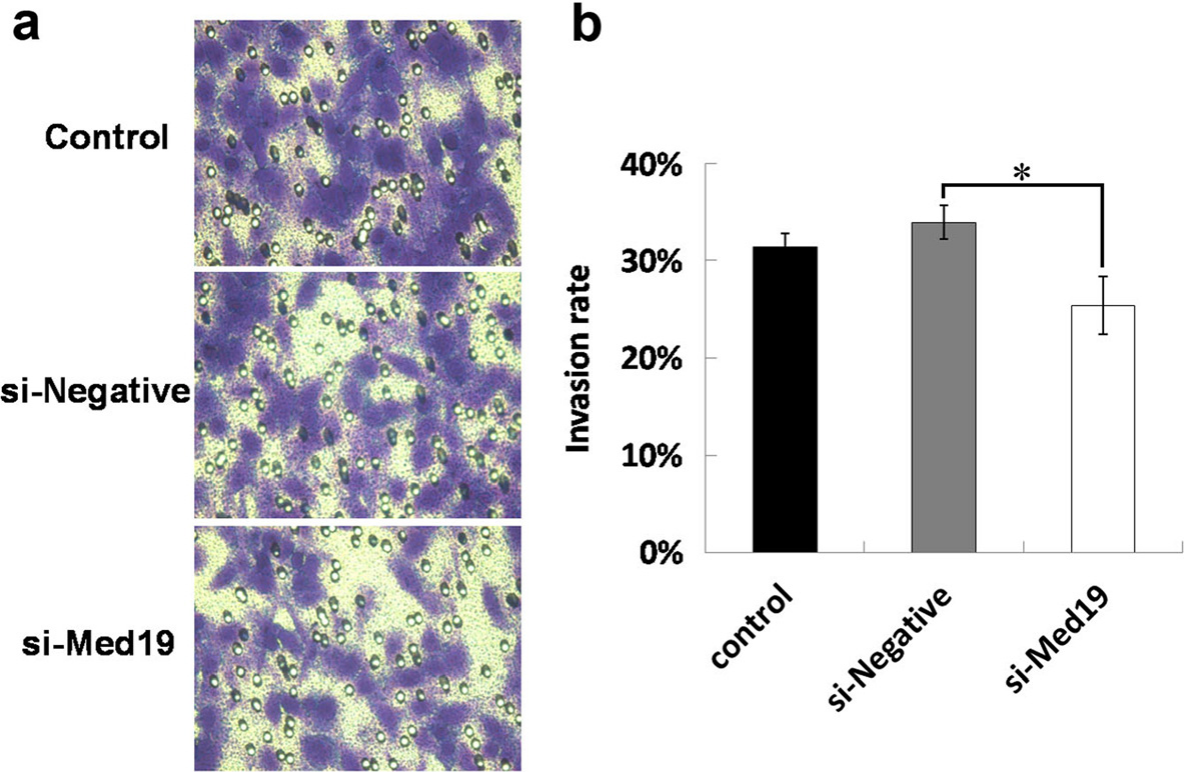
**Med19 shRNA inhibit tongue cancer cell migration.** (**a**) Pictures of crystal violet staining cells. (**b**) Med19 shRNA treated cells show a decrease in the number of migrated cells. Mean values are derived from three independent experiments. Errors indicate standard deviation.

To test whether tongue cancer cell migration was affected by Med19 shRNA, a transwell assay was performed. As showed in Figure 3a, when treated with Med19 shRNA, tongue cancer cell migration was inhibited. The migration rate was 31%, whereas in the Med19-knockdown group, it was only 25% (Figure 3b, *P* <0.05). In this way, we could conclude that knockdown of Med19 could inhibit cell migration of tongue cancer cells. Med19 possibly plays a critical role in the migration of tongue cancer.

### The *in vivo* tumor growth of tongue cancer cells is inhibited by Med19 shRNA

**Figure 4:**
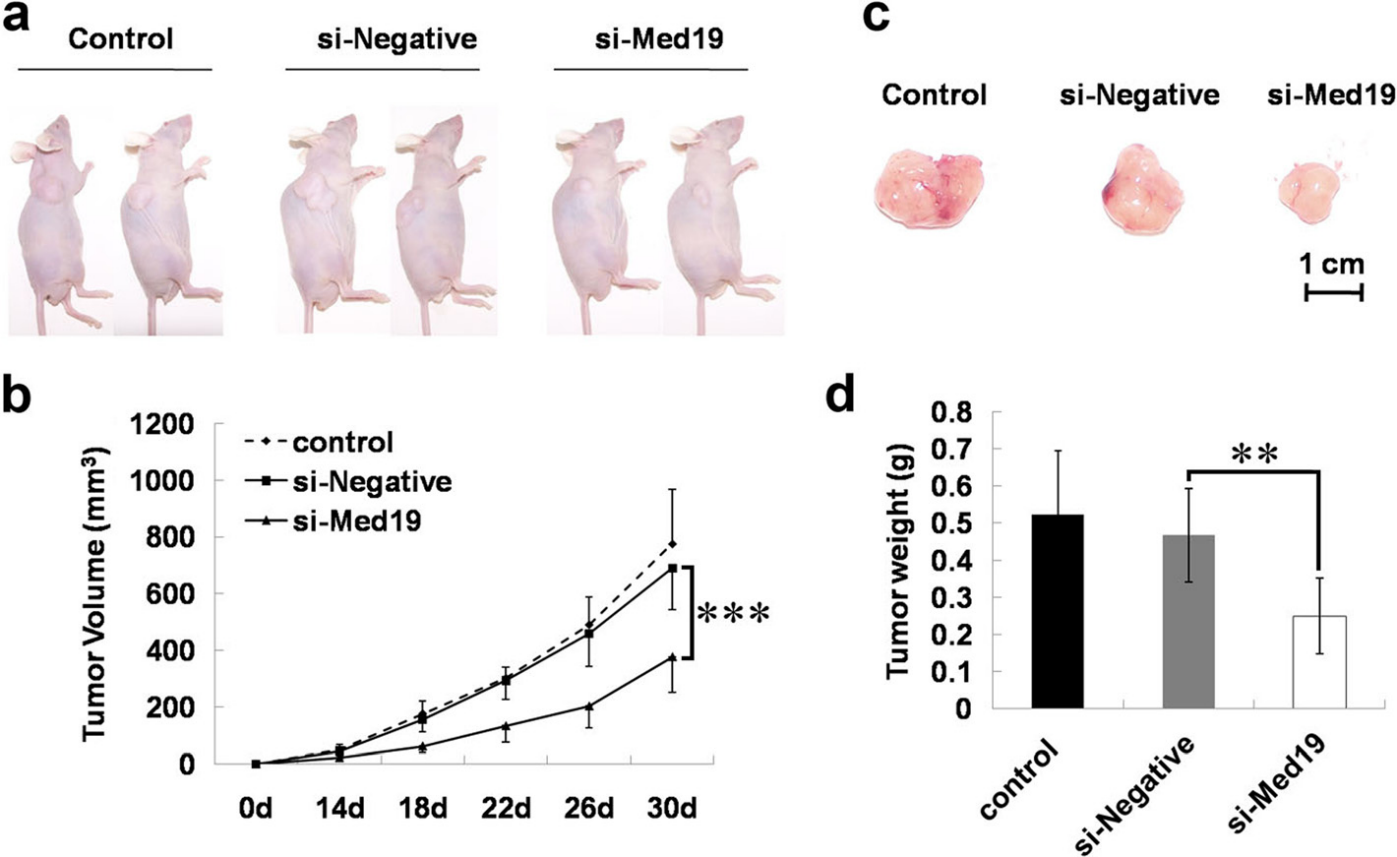
**Med19 shRNA inhibits tongue cancer cell tumor growth in *vivo*.** Representative images of tongue tumor-bearing mice (**a**) and tumors from mice (**c**). (**b**) Med19 shRNA treated tongue cancer cells show smaller tumor volume. (**d**) Med19 shRNA treated tongue cancer cells show lower tumor weight.

To further explore the tumor inhibited ability of Med19 shRNA, we used a xenograft model to examine whether Med19 shRNA inhibited tumor growth *in vivo* (Figure 4a). When inoculated subcutaneously into athymus nude mice, tongue cancer cells treated with Med19 shRNA had dramatically reduced tumor volumes (Figure 4b and 4c) and tumor weights (Figure 4d) compared with blank control cells (Control) and control negative shRNA treated with cells, indicating that Med19 promotes the tumorigenesis of tongue cancer cells.

## Discussion

Tongue carcinoma is one of the most common types of oral cancer [2], which represents the sixth most common cancer around the world [1]. Despite the improvements in treatment for tough cancer, the etiology of this disease is still unknown. With the help of lentivirus-mediated gene silencing technique, more and more genes have been implicated to be associated with carcinogenesis in recent years. Among these cancer-related genes, many gene expression patterns have been demonstrated to be altered partially due to transcriptional regulation. Thus, elucidation of critical roles of transcriptional regulators will help us to understand the progression of tongue carcinogenesis.

Med19 is one of the components of the Mediator complex, which acts as a molecular bridge between transcriptional activator and RNA polymerase II [17]. Although Med19 has been suggested to play an important role in many cancer types [11-15], the pathologic importance of this molecule in tongue cancer is yet unknown. In this study, we investigated the antitumorigenic effects of lentivirus-mediated transduction of Med19 shRNA into tongue cancer cells by quantifying cellular proliferation, tumor growth, cell cycle and migration ability. Our results suggest that downregulation of Med19 by shRNA resulted in inhibition of cell proliferation, colony formation and migration ability, and induction of G0/G1 phase cell cycle arrest. Furthermore, the antitumor effects of Med19 were elucidated by *in vivo* tumorigenicity experiments. Our research was the first report in which it has been demonstrated that Med19 modulates tongue cancer cell proliferation and migration and disruption of Med19 has antitumorigenic effects.

The mechanism through which Med19 induced tongue carcinogenesis is still unknown. It has been reported that many components of the Mediator complex can directly bind a variety of regulatory transcription factors that are indispensible for cell growth and differentiation. For example, *MED1/TRAP220* and *MED17* can bind to GATA family membranes [18], *BRCA-1*[19] and *P53*[20]. Thus, it is possible that there may be interactions between Med19 and these cell growth transcription factors via direct or indirect binding. Udayakumar and his colleagues have showed that the Mediator complex regulates gene expression of Aurora-A, a centrosome kinase critical for cell cycle progression [21]. In prostate cancer, it has been demonstrated that the expression of *CDK4* was decreased after *Med19* disruption [15]. Overall, although it is possible that Med19 like other components of the Mediator complex, could directly or indirectly bind with transcription factors, which are necessary for cell growth and cell cycle progression, it is still worth working to demonstrate the exact signaling pathway involved in the regulation of progression in tongue cancer by Med19.

RNA interference (RNAi) is sequence-specific post-transcriptional gene silencing by short small interfering RNA (siRNA), which is thought to be a powerful approach for studying gene function and gene therapy [22]. Due to the ease of delivery, adenovirus, retrovirus, and lentivirus are commonly used for shRNA transduction [23-25]. Lentiviruses are considered to be more suitable for gene therapy for their safety and life-long expression of shRNA [26,27].

## Conclusions

Our results provide the evidence that lentivirus-mediated Med19 downregulation inhibits tongue cancer cell proliferation and tumorigenesis both *in vitro* and *in vivo*, suggesting that disruption of Med19 by lentivirus transduction may be a promising approach for tongue cancer therapy.

## Abbreviations

Med19: Mediator complex subunit 19; FACS: Fluorescence-activated cell sorting; MOI: Multiplicity of infection; PVDF: Polyvinylidene difluoride; RT-PCR: Reverse transcription polymerase chain reaction; shRNA: Short hairpin RNA.

## Competing interests

The authors declare that they have no competing interests.

## Authors’ contributions

All authors read and approved the final manuscript.
